# An immunoinformatics approach to epitope-based vaccine design against PspA in *Streptococcus pneumoniae*

**DOI:** 10.1186/s43141-023-00506-9

**Published:** 2023-05-11

**Authors:** Lincon Mazumder, Muhammad Shahab, Saidul Islam, Mahmuda Begum, Jonas Ivan Nobre Oliveira, Shamima Begum, Shahina Akter

**Affiliations:** 1 Department of Microbiology, Jagannath University, Dhaka, 1100 Bangladesh; 2 State Key Laboratories of Chemical Resources Engineering, Beijing University of Chemical Technology, Beijing, 100029 China; 3 Bangladesh Council of Scientific & Industrial Research (BCSIR), Dhaka, 1205 Bangladesh; 4 Departamento de Biof ´ısica E Farmacologia, Universidade Federal Do Rio Grande doNorte, Natal, RN 59072-970 Brazil

**Keywords:** SPN, pneumococcal surface protein A (PspA), CTL, HTL, LBL, TLR4

## Abstract

**Background:**

*Streptococcus pneumoniae* (SPN) is the agent responsible for causing respiratory diseases, including pneumonia, which causes severe health hazards and child deaths globally. Antibiotics are used to treat SPN as a first-line treatment, but nowadays, SPN is showing resistance to several antibiotics. A vaccine can overcome this global problem by preventing this deadly pathogen. The conventional methods of wet-laboratory vaccine design and development are an intense, lengthy, and costly procedure. In contrast, epitope-based in silico vaccine designing can save time, money, and energy. In this study, pneumococcal surface protein A (PspA), one of the major virulence factors of SPN, is used to design a multi-epitope vaccine.

**Methods:**

For designing the vaccine, the sequence of PspA was retrieved, and then, phylogenetic analysis was performed. Several CTL epitopes, HTL epitopes, and LBL epitopes of PspA were all predicted by using several bioinformatics tools. After checking the antigenicity, allergenicity, and toxicity scores, the best epitopes were selected for the vaccine construction, and then, physicochemical and immunological properties were analyzed. Subsequently, vaccine 3D structure prediction, refinement, and validation were performed. Molecular docking, molecular dynamic simulation, and immune simulation were performed to ensure the binding between HLA and TLR4. Finally, codon adaptation and in silico cloning were performed to transfer into a suitable vector.

**Results:**

The constructed multi-epitope vaccine showed a strong binding affinity with the receptor molecule TLR4. Analysis of molecular dynamic simulation, C-immune simulation, codon adaptation, and in silico cloning validated that our designed vaccine is a suitable candidate against SPN.

**Conclusion:**

The in silico analysis has proven the vaccine as an alternative medication to combat against *S. pneumoniae*. The designated vaccine can be further tested in the wet lab, and a novel vaccine can be developed.

**Supplementary Information:**

The online version contains supplementary material available at 10.1186/s43141-023-00506-9.

## Background

*Streptococcus pneumoniae* (SPN) is the etiological agent of most of the community-acquired pneumonia and the reason behind millions of deaths worldwide [1–3]. This elongated round-shaped, gram-positive, alpha-hemolytic, encapsulated, nonmetals, non-flagellated, nonspore-forming bacterium can be found in the human respiratory tract as a commensal organism [4, 5]. This pathogen can asymptomatically colonize the nasopharynx, and over time, it can migrate to other cells and cause infections in sterile tissues and organs [1]. However, pneumococcus infection may give rise to severe health concerns by causing not only pneumonia but also bronchitis, brain abscess, otitis media, septicemia, meningitis, osteomyelitis, cellulitis, pericarditis, endocarditis, conjunctivitis, peritonitis, and acute sinusitis [1, 4, 6]. Although pneumococcus by SPN can occur in any group, it is more prevalent in individuals over the age of 65 and children under the age of 2 [7]. SPN is the most prevalent infectious agent causing mortality in infants under the age of 5 [8]. In 2015, approximately 335,000 deaths (with a range of 240–460,000) in children (< 5 years) have been reported globally due to pneumococcal infection [9]. According to the European Centre for Disease Prevention and Control, a total of 24,663 confirmed cases of invasive pneumococcal disease (IPD) were recorded throughout the EU/EEA in 2018. In the USA, the frequency of IPD was reported to be 7 per 100,000 people nationwide in 2018 as per the report of the Centers for Disease Control and Prevention (CDC). The overall mortality rate in the children (< 59 months) of the South African region was estimated to be 36 per 100,000 children between the year 2012 to 2013 [10]. According to the World Health Organization (WHO) data, approximately 113,000 deaths from pneumococcal pneumonia and pneumococcal meningitis were reported in South Asia in 2008 [11].

Though antibiotics are traditionally used as a first-line treatment for pneumococcal infections, several dispensable gene-mediated resistance to antibiotics is growing rapidly in some strains of SPN and therefore losing their reliability for the treatment of pneumococcal diseases [1, 2]. The use of an effective vaccine may help to prevent this disease in most cases. Although currently a number of pneumococcal vaccines are available in the market, most of them have several shortcomings, including elevated toxicity, poor solubility, lower protection, complex composition, difficulty to manufacture, and risk of inducing allergy (skin rash). Two types of vaccine, including protein-conjugated polysaccharide vaccines (PCV13, PCV15, and PCV20) and unconjugated (plain) polysaccharide vaccines (PPSV23), are the available forms of pneumococcal vaccine until recently. According to the recommendation of CDC, PCV13 and PCV15 are most commonly used in the USA for vaccinating babies and children younger than 5 years old. PCV15, when applied to adults of 65 years or older, need to be followed by another dose of PPSV23. However, the conjugated pneumococcal vaccine (PCV) may shield children from pneumococcal infections, but it is ineffective in older persons (> 65 years) who can develop pneumococcal diseases even after receiving the vaccine since the vaccine can exclude the serotypes associated with the disease [12–14]. Similarly, the unconjugated polysaccharide vaccine (PPSV), which incorporates T-cell independent polysaccharide antigens, is ineffective against the pathogen’s greatest risk group—infants under the age of 2 [12]. Besides, the increasing events of antimicrobial resistance among disease-causing microorganisms unequivocally highlight the necessity to develop a new strategy or new vaccine to combat pneumococcal infections in the early stage. Creating a new vaccine in a cost-effective way that can give better protection against this pathogen without any possible side effects can be a viable solution to overcome the shortcomings of currently available vaccines.

Currently, a number of vaccine development strategy is available. Inactivation or live attenuation of the pathogen is one of the traditional methods for vaccine development. Although vaccines developed in this process can show a strong immune response in the host, these can also produce undesirable allergic and toxic reactions in the host. Similarly, several recombinant vaccines including conjugated vaccines, subunit vaccines, and toxoid vaccines may also produce severe toxicity instead of eliciting desired immune response [15]. In contrast, an epitope-based vaccine has been demonstrated to be safe and does not cause any immunological problems in the host [16]. Through the immense revolution of computerized biology, it is now easier to design an epitope-based vaccine with dry-lab experiments, which can be further verified with wet-lab confirmatory experiments [17]. Dry-lab experiments which are also known as in silico experiments may reduce the time and cost of wet lab experiments and thus becoming a site of interest among researchers. However, the main challenge of in silico vaccine construction is that the in silico analysis is not fully reliable until wet laboratory experiments verify it. Analysis with computational databases may result in spurious outcomes due to their limitations. Hence, vaccines produced by the in silico experiment must be tested in vitro, followed by clinical trials in larger animals, to ensure their efficacy. The main aim of this study is to design a novel multi-epitope vaccine to combat pneumococcal infection. Thus, throughout this research, we will investigate a possible epitope-based vaccine candidate by using an immunoinformatics approach and finally design a multi-epitope vaccine by in silico process.

Several virulence factors of SPN may influence its spread in the host cell, escape the immune defense systems, and promote disease progression [18]. Polysaccharide capsule, pneumolysin, autolysin, pneumococcal surface protein A, pneumococcal surface protein C, pneumococcal surface adhesin A, neuraminidase, and several other choline-binding proteins are some of the virulence proteins of *S. pneumoniae* which are currently known [1, 6]. Several in silico studies have been performed and reported before to evaluate these virulence proteins as vaccine candidates for epitope-based vaccine design against SPN. Munia et al. (2021) evaluated the pneumococcal choline-binding protein A (CbpA) to design an epitope-based vaccine [19]. In contrast, Tarahomjoo and Ghaderi (2017) studied choline-binding protein D (CbpD) and *Salmonella enteritidis* flagellin as vaccine candidates [20]. Tarahomjoo and Ghaderi (2019) also reviewed fibronectin-binding protein (FBP) and D-alanyl-D-alanine-carboxypeptidase (DDCP) as vaccine candidates [21]. Dorosti, Eslami, and Negahdaripour et al. (2019) used a mixture of pneumococcal surface protein A (PspA), choline-binding protein A (CbpA), pneumococcal histidine triad D (PhtD), and pneumococcal iron uptake protein (PiuA) to design a multi-epitope peptide pneumococcal vaccine [22]. In another study, Dorosti et al. (2019) also evaluated the PspA, CbpA, and PiuA for developing a peptide nanovaccine [14]. Pneumococcal surface proteins A and C (PspA and PspC) and pneumococcal histidine triad D (PhtD) were investigated as potential epitope-based vaccine candidates against SPN by Bahadori et al. (2022) [12].

Pneumococcal surface protein A (PspA) is one of the important virulence factors which is found on the surface of SPN [3]. Mutant strains of SPN which lack PspA protein have shown to have lower virulence in the asepsis model [23]. PspA is known to involve with the inhibition of opsonization, neutralization of several antimicrobial factors, or serving as adhesins, independently [24]. PspA has previously been reported to bind to lactoferrin [5] and interferes with the complement system [4, 25]. By aiding the colonization and decreasing the deposition of the complement system, PspA is known to promote the virulence of the bacteria [1]. Because of its direct role in pathogenesis, we considered PspA as a suitable target antigen of SPN for multi-epitope vaccine design.

## Methods

**Fig. 1:**
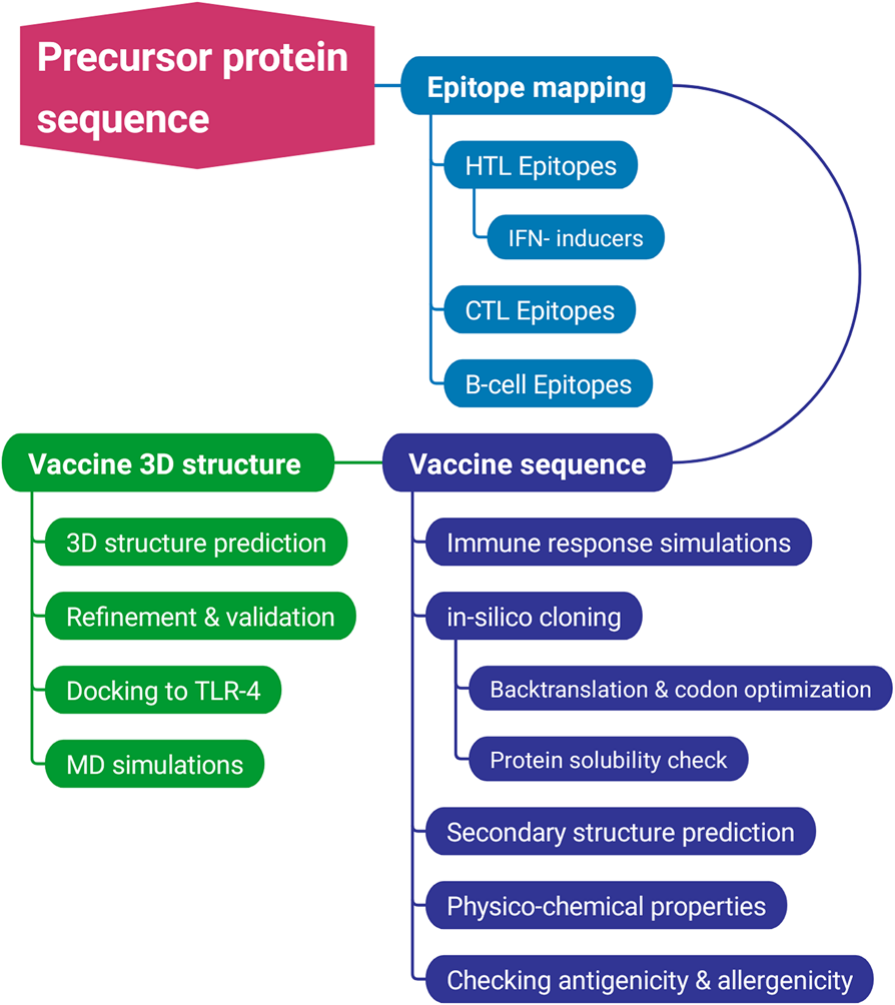
Workflow and tools used in this study for in silico design of multi-epitope vaccine

The complete workflow and various tools used in this study for designing a multi-epitope vaccine by in silico processes are depicted in Fig. 1.

### Sequence retrieval and prioritization

The reference sequence of the PspA (accession no.: WP_001035315.1) was first retrieved from NCBI and then subjected to protein–protein blast (Blastp) [26] against the nonredundant database, and the top 10 sequences (including the reference sequence) were retrieved as FASTA format. Multiple sequence alignment was conducted by the MUSCLE v3.6 program [27]. Phylogenetic analysis of all the 10 sequences was performed using Mega X [28]. Sequence prioritization was performed to identify the best sequence as the vaccine target based on its non-allergen property and ability to be an antigen. Antigenicity and allergenicity of all the retrieved sequence were determined by using VaxiJen v2.0 (http://www.ddg-pharmfac.net/vaxijen/VaxiJen/VaxiJen.html) [29] and AllerTOP v2.0 (https://www.ddg-pharmfac.net/AllerTOP/) [30] server, respectively. The threshold parameter of the VaxiJen server was set to 0.4, throughout this study. The protein with the highest antigenicity and non-allergen property was selected for further analysis.

### Prediction of CTL epitope

NetCTL v1.2 (https://services.healthtech.dtu.dk/service.php?NetCTL-1.2) [31] was used to predict the cytotoxic T lymphocytes of the chosen protein sequence. The parameter of the threshold was set to 0.4 to obtain 0.89 sensitivity and 0.94 specificity, and among the 12 supertypes (A1, A2, A3, A24, A26, B7, B8, B27, B39, B44, B58, and B62) of MHC class 1, only A1 supertype was selected within the NetCTL parameters. The identified epitopes were further evaluated with VaxiJen v2.0, ToxinPred (https://webs.iiitd.edu.in/raghava/toxinpred/multi_submit.php) [32], and AllerTop v2.0 with default parameters to investigate the antigenic, allergenic, and toxicity properties of the protein sequence, respectively.

### Prediction of HTL epitope

Helper T lymphocytes (HTL) were identified by utilizing the MHC II search tool of the “Immune Epitope Database (IEDB)” (http://tools.iedb.org/mhcii/) [33]. The NN-align method and the complete set of alleles were selected during the prediction of HTL. VaxiJen v2.0, AllerTop v2.0, and IFNepitope analysis were further carried out to identify the 3 best epitopes for vaccine preparation. IFNepitope server (https://webs.iiitd.edu.in/raghava/ifnepitope) [34] was used for the analysis of the IFN-*γ* response.

### Prediction of LBL epitope

Linear B-cell lymphocyte (LBL) epitopes were predicted by using the IEDB Kolaskar and Tongaonkar antigenicity method (http://tools.iedb.org/bcell/). This tool identifies LBL by utilizing a semiempirical method for the prediction of antigenic determinants on protein antigens. The amino acid sequences of the selected protein were submitted in this tool, and best 3 epitopes were selected for vaccine construction.

### Formulation of multi-epitope vaccine

Vaccine construction becomes successful when it can stimulate both innate and adaptive immunity. For stimulating both innate and adaptive immunity, CTL epitopes, HTL epitopes, and LBL epitopes are used [35]. To make more strong immune stimulation, adjuvant is used. 50 s ribosomal protein is used as an adjuvant fused with the CTL epitopes by “EAAAK” linker [36]. CTL epitopes joined each other by “AAY” linker, enhancing the epitopes presentation. The “GPGPG” linker links CTL epitopes and HTL epitopes. “GPGPG” linkers also join HTL epitopes. Furthermore, the “KK” linker joins the HTL epitopes and LBL epitopes. Linkers help to stimulate immunity by producing higher antibody titers [37].

### Physicochemical and immunological properties analysis

The physiochemical properties of the vaccine were evaluated with the ExPasy ProtParam server [38], which can be found at https://web.expasy.org/protparam/, and several essences of the vaccine including the number and composition of amino acid residues, molecular weight, number of positively and negatively charged residues, grand average of hydropathicity (GRAVY), theoretical pI, and aliphatic and instability index were comprehended from this server. Subsequently, VaxiJen v2.0, MHC-I immunogenicity, AllerTop, ToxinPred, and SOLpro (http://scratch.proteomics.ics.uci.edu/) [39] servers were utilized for evaluating the vaccine’s immunological properties.

### Vaccine 3D structure prediction, refinement, and validation

The three-dimensional structure of the vaccine was constructed by using SCRATCH server (http://scratch.proteomics.ics.uci.edu/) [40]. This server predicts the 3D structure by simply utilizing an amino acid sequence. For the refinement of the predicted 3D structure, GalaxyRefine server (http://galaxy.seoklab.org/refine) [41] was used. PROCHECK’s Ramachandran plot [42] and ERRAT [43] from the SAVES server (https://saves.mbi.ucla.edu/) were utilized for structure validation.

### Molecular docking

Molecular docking is the most powerful computational tool that predicts the interaction between protein–protein and protein ligand. To ensure the binding between HLA molecules TLR4 and our predicted vaccine, a docking study was performed using ClusPro 2.0 (https://cluspro.bu.edu/) [44], an online-based docking tool. All the docking parameters in this tool were kept as default during the analysis. The structure of receptor TLR4 was downloaded from the RCSB protein database (PDB) [45]. The receptor, TLR4 molecule, was subsequently prepared for docking by removing associated ligand groups, water molecules, and other chemicals with BIOVIA Discovery studio 2021 [46]. The interaction in the docked complex was visualized by utilizing PyMOL 2 software [47]. The interacting residues in the docked complex were further investigated with PDBsum tool [48].

### MD simulation

To understand the residual impact of the vaccine with the receptor (TLR-4) consistently evoke an immune response against target cells. MD simulation was performed for TLR4-vaccine complex using iMods online server (https://imods.iqfr.csic.es/) [49]. These tools involve the exploration of macromolecule for molecular structure exploration. This server provides different analyses, i.e., NMA mobility calculation, deformability, eigenvalue, and B-factor can be calculated using this tool.

### C-immune prediction

C-immune tool predictor (https://kraken.iac.rm.cnr.it/C-IMMSIM/) [50] position-specific scoring matrix that is used to understand immune response magnitude which showed the result of vaccine dosage concerning different time intervals.

### Codon adaptation and in silico cloning

EMBOSS backtranseq (https://www.ebi.ac.uk/Tools/st/emboss_backtranseq/) [51] was used to obtain the DNA sequence from the protein sequence of the constructed vaccine. Subsequently, the JCat server (http://www.jcat.de/) [52] was used for the optimization of the DNA sequence to adapt its codon to most sequenced prokaryotic organisms (*Escherichia coli* K12). GC content and CAI value were measured for the adapted and unadapted sequences. The presence of restriction sites in the vaccine construct was investigated in order to clone it to a suitable vector. Finally, the codon-optimized (adapted) DNA sequence of the vaccine was cloned into the *E. coli* pET28a( +) vector using the SnapGene® tool (from Insightful Science; available at https://snapgene.com).

## Results

### Sequence retrieval, phylogenetic analysis, and sequence prioritization

**Fig. 2:**
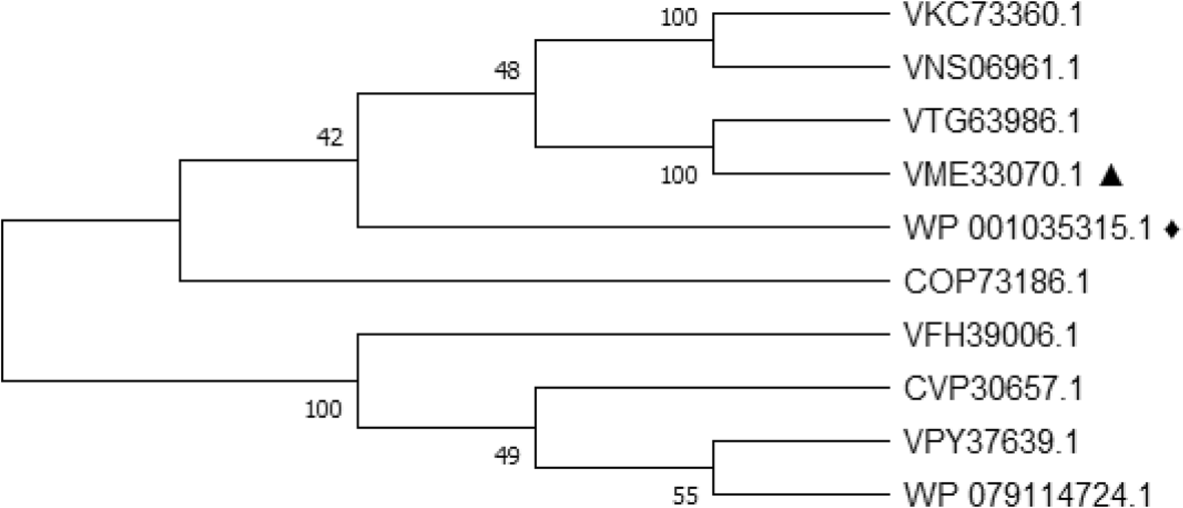
Phylogenetic relationship among the studied protein (marked with ▲ symbol), reference protein (marked with ♦ symbol), and other proteins obtained from nonredundant database by BlastP search. The evolutionary distances were computed using the Poisson correction method and are in the units of the number of amino acid substitutions per site

Protein information of the top 10 protein sequences, obtained by BlastP against the nr database, is depicted in Supplementary Table 1, with their properties including antigenicity, allergenicity, and toxicity. MUSCLE v3.6 method was performed for multiple sequence alignment. A phylogenetic tree showing the phylogenetic relatedness among the sequences was constructed using the MEGA X program by neighbor-joining method [53] with a bootstrap replication of 1000, shown in Fig. 2. After analyzing these 10 sequences, protein sequence with accession number VME33070.1 was found to be the most potent antigenic protein with a VaxiJen score of 0.6675. AllerTOP server also declared this sequence as a non-allergen protein. Analysis with VaxiJen and AllerTOP thus indicates the protein as a suitable vaccine target. Hence, the protein with accession number VME33070.1 was selected in this study to design a multi-epitope-based vaccine.

### Prediction of CTL epitope

Currently used vaccines mostly rely on B-cell immunity. Antigenic drift eventually allows any foreign substance to escape the antibody memory response. As a result, multi-epitope vaccines have been promoted because they can provide long-lasting protection when B cells, T-helper cells (CD4 +), and cytotoxic T cells (CD8 +) are combined. The host’s CD8 + T cells are capable of generating a potent immune response that targets the infected cell [54]. Hence, cytotoxic T cell was identified in the sequence of interest with NetCTL 1.2 server. A total number of 14 cytotoxic T lymphocytes (CTLs) were predicted from NetCTL 1.2 server, and best three CTLs were screened out for vaccine construction. Table 1 depicts the best three epitopes along with their C-score, antigenicity score, immunogenicity score, toxicity, and allergenic properties. All 14 epitopes identified from NetCTL 1.2 server are given in Supplementary Table 2.

**Table 1:** CTLs identified in the sequence by using NetCTL 1.2 server

Peptide	C-score	Antigenicity	Immunogenicity	Toxicity	Allergenicity
AMDEAEKEY	1.7329	0.4003	0.1168	Negative	Negative
KSEAAKKEY	1.643	1.5677	− 0.21653	Negative	Negative
KSEAAKKHY	1.5182	1.4407	− 0.25613	Negative	Negative

### Prediction of HTL epitope

Identifying helper T cells is essential during the design of a multi-epitope vaccine. T-helper cells (CD4 +) play a crucial role in inhibiting immunological response and controlling the efficient immune response to pathogens. In addition to these functions, T-helper cells also activate innate immune system cells, B lymphocytes, and cytotoxic T cells [55]. Among 155 epitopes with ic50 less than 250, predicted with IEDB MHC II search tools, 26 epitopes (Supplementary Table 3) were shortlisted based on their interaction with at least 10 alleles. Among these 26 epitopes, 3 epitopes were selected for vaccine preparation. These 3 epitopes were found to be the most antigenic epitopes with non-allergen and nontoxic properties, depicted in Table 2.

**Table 2:** Predicted three HTL with their antigenic score and allergenic and toxic properties

Epitopes	Interacting alleles	Antigenicity	Allergenicity	Toxicity	IFN-*γ* (score)
TWYYLEASGAMKASQ	HLA-DQA1*05:01/DQB1*03:01, HLA-DQA1*01:02/DQB1*06:02, HLA-DRB1*07:01, HLA-DRB1*13:02, HLA-DRB1*01:01, HLA-DRB5*01:01, HLA-DRB1*04:05, HLA-DRB1*11:01, HLA-DRB1*09:01, HLA-DRB1*04:01, HLA-DRB3*01:01	0.7340	Negative	Negative	Positive 0.69662678
SWYYLNANGAMATGW	HLA-DRB3*02:02, HLA-DQA1*01:02/DQB1*06:02, HLA-DRB1*13:02, HLA-DQA1*05:01/DQB1*03:01, HLA-DRB5*01:01, HLA-DRB1*01:01, HLA-DRB1*11:01, HLA-DRB1*04:05, HLA-DRB1*04:01, HLA-DRB1*09:01, HLA-DRB3*01:01, HLA-DRB1*07:01	0.6068	Negative	Negative	Positive 1.3611371
GSWYYLNANGAMATG	HLA-DRB1*13:02, HLA-DQA1*01:02/DQB1*06:02, HLA-DQA1*05:01/DQB1*03:01, HLA-DRB1*04:05, HLA-DRB5*01:01, HLA-DRB1*15:01, HLA-DRB1*01:01, HLA-DRB1*11:01, HLA-DRB3*02:02, HLA-DRB1*04:01, HLA-DRB1*09:01, HLA-DRB3*01:01, HLA-DRB1*07:01	0.6041	Negative	Negative	Positive 0.29956413

### Prediction of LBL epitope

Due to its capability to produce antibodies that interact with antigens, B-cell epitope identification in target antigens has gained immense interest among researchers during the development of multi-epitope-based vaccines. An effective and promising method for locating possible B-cell epitopes in a target vaccination candidate is to use in silico bioinformatics techniques [56]. In total, 21 B-cell epitopes (Supplementary Table 4) were identified from the IEDB web server, and the top three epitopes (Table 3) were selected based on their antigenicity, allergenicity, and toxicity properties. The nonantigenic, allergenic, and toxic epitopes predicted by VaxiJen, AllerTop V 2.0, and ToxinPred respectively were eliminated from the study.

**Table 3:** LBL epitopes predicted with IEDB B-cell epitope prediction tool by utilizing Kolaskar and Tongaonkar antigenicity method

No	Start	End	Peptide	Length	Antigenicity	Allergenicity	Toxicity
2	42	48	DAAVKKS	7	1.7312	Negative	Non-toxin
14	461	466	WYYLNA	6	1.1522	Negative	Non-toxin
20	601	607	WYYLEAS	7	1.0143	Negative	Non-toxin

### Formulation of multi-epitope vaccine

**Fig. 3:**
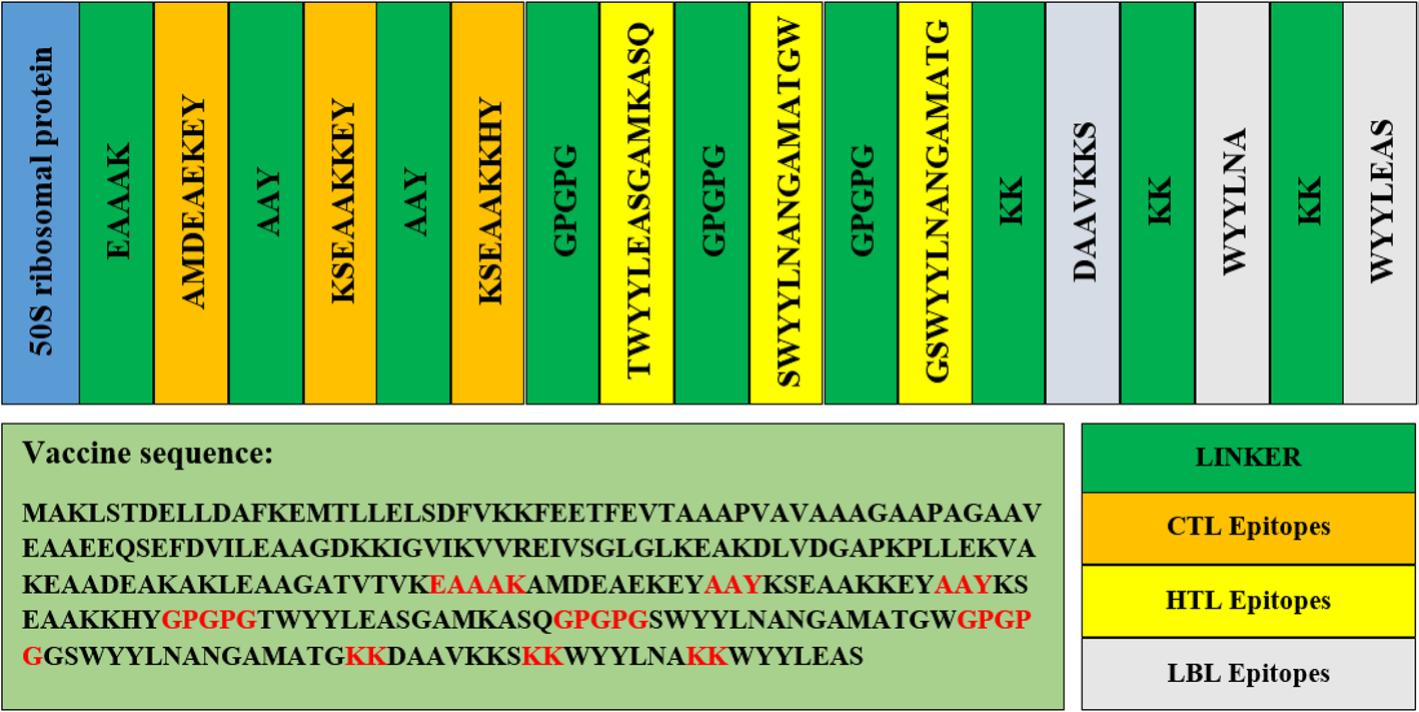
Formulation of multi-epitope vaccine by combining adjuvant, linkers, CTL epitopes, HTL epitopes, and LBL epitopes

Epitope prediction was followed by the formulation of vaccine. The epitopes for vaccine formulation were determined on the basis of checking the antigenic property of both B and T cells. Using VaxiJen v2.0, the ACC calculation of the peptide based on its physicochemical properties was performed. Three best epitopes that have the highest antigenic score and nonallergenic and nontoxic properties were screened from each set of the CTL, HTL, and LBL epitopes. For the formulation of the vaccine construct, 50 s ribosomal protein, which is used as an adjuvant, is attached with the CTL epitopes by “EAAAK” linker. The CTL epitopes are connected to each other by the “AAY” linker. As three CTL epitopes are used in this study, two “AAY” linkers have been used. CTL epitopes and HTL epitopes are connected by the “GPGPG” linker, and HTL epitopes are also linked among themselves with the “GPGPG” linker. For this reason, three “GPGPG” linkers are used. The next portion is LBL epitopes, which are connected to the HTL epitopes by the “KK” linker, and LBL epitopes themselves are also connected by the “KK” linker. So, three “KK” linkers are required to formulate the vaccine. The complete procedure of formulating the multi-epitope vaccine is illustrated in Fig. 3.

### Physicochemical and immunological properties analysis

Physicochemical properties of the vaccine construct are depicted in Table 4. The ProtParam server identified that the constructed vaccine consists of a total number of 254 amino acids, with 36 and 33 as negatively charged residues (Asp + Glu) and positively charged residues (Arg + Lys), respectively. The molecular weight was calculated as 26,817.46, whereas the theoretical isoelectric point (pI) was found to be 5.54. The instability index was computed to be 20.34, which classifies the protein as a stable one. The grand average of hydropathicity (GRAVY) score was found to be − 0.303. Among the amino acid composition, alanine was found to be more prominent (21.3%), which was followed by lysine (12.6%), glutamate (10.2%), glycine (9.1%), leucine (7.1%), tyrosine and valine (5.9%), serine (4.7%), proline and aspartate (3.9%), threonine (3.5%), tryptophan and methionine (2.4%), phenylalanine and asparagine (2.0%), isoleucine (1.6%), glutamine (0.8%), and arginine and histidine (0.4%). The VaxiJen v2.0 server predicted the antigenic score of the vaccine construct as 0.5722 which classified the protein as an antigenic protein. The vaccine construct sequence was defined as non-allergen by AllerTop v2.0 server further verified its suitability for vaccine construction. SOLpro defined the sequence as soluble with a probability score of 0.919.

**Table 4:** Physicochemical properties of the protein identified with ExPasy ProtParam server

Amino acid composition (number of residues)	Ala (54), Arg (1), Asn (5), Asp (10), Gln (2), Glu (26), Gly (23), His (1), Ile (4), Leu (18), Lys (32), Met (6), Phe (5), Pro (10), Ser (12), Thr (9), Trp (6), Tyr (15), Val (15)
Total number of amino acids	254
Molecular weight	26,888.54
Theoretical pI	5.54
Total number of negatively charged residues (Asp + Glu)	36
Total number of positively charged residues (Arg + Lys)	33
Instability index	The instability index (II) is computed to be 20.34. This classifies the protein as stable
Aliphatic index	72.17
Grand average of hydropathicity (GRAVY)	− 0.303

### Vaccine 3D structure prediction, refinement, and validation

**Fig. 4:**
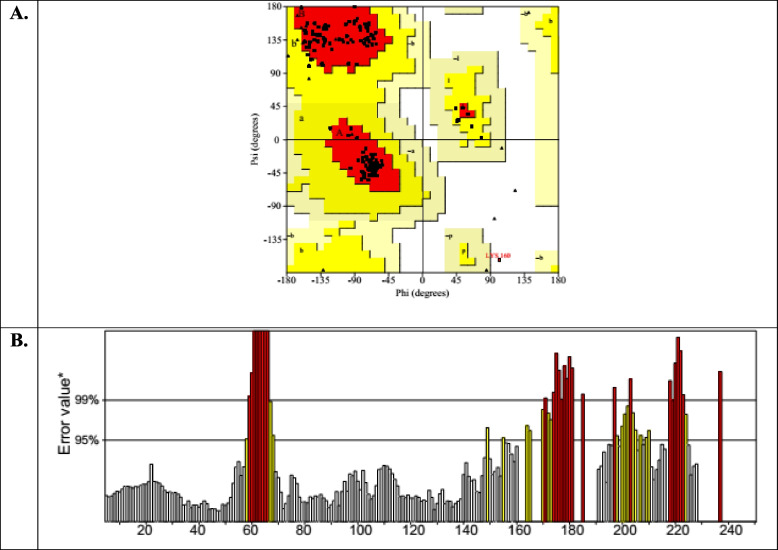
3D model (refined) of the predicted vaccine validated by Ramachandran plot (**A**) of PROCHECK program and ERRAT (**B**) of SAVES server

**Fig. 5:**
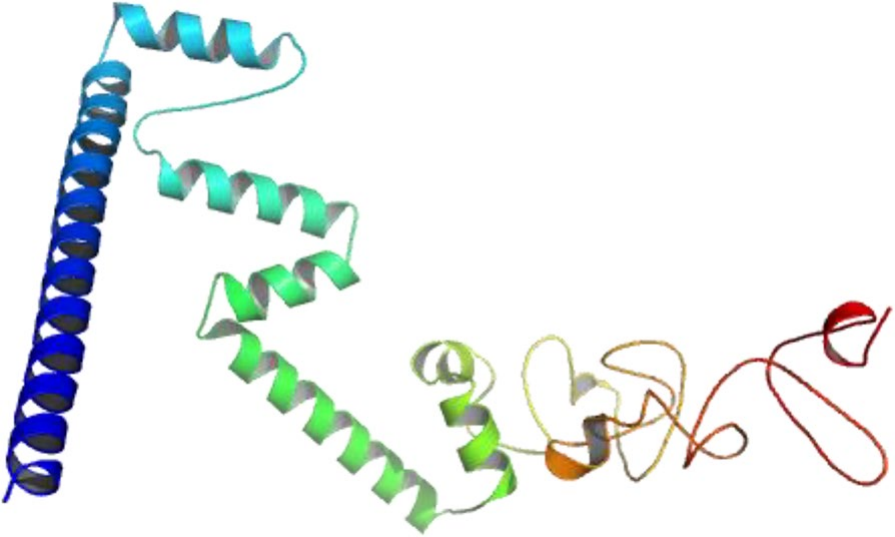
Vaccine 3D structure rendered with PyMOL 2

A three-dimensional (3D) structure of the protein from vaccine sequence was obtained from SCRATCH server. GalaxyRefine server was subsequently utilized for the refinement of the protein 3D structure. GalaxyRefine server made the protein structure more stable and increased its quality score in SAVES server. GalaxyRefine server gave 5 refined models of the protein structure, and among these models, model 2 was selected as the best model on basis of its quality score. Ramachandran plot analysis has revealed that 95.4% residues of the 3D structure were in the most favored region for the refined structure (Fig. 4A). Before the refinement. this score was 91.8%. Similarly, the overall quality factor in the ERRAT program was increased to 77.512 from the initial value 56.9106, upon the refinement (Fig. 4B). The 3D model of the refined vaccine structure was visualized and rendered with PyMOL 2 (Fig. 5).

### Molecular docking studies between the vaccine construct and TLR4 receptor

**Fig. 6:**
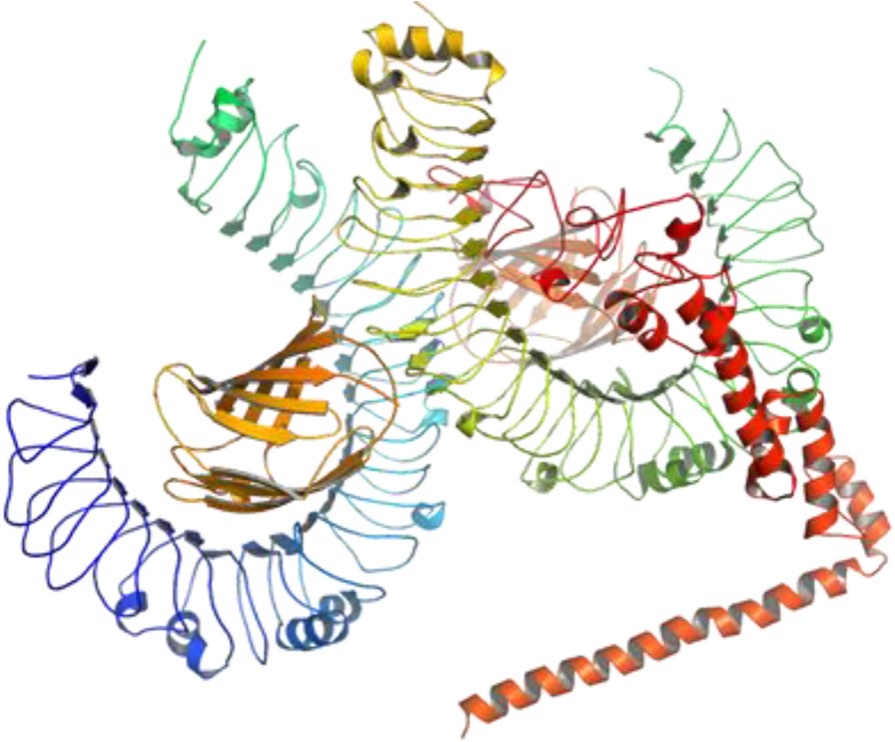
Molecular docking interaction between the vaccine construct and TLR4 receptor illustrated with PyMOL 2

**Fig. 7:**
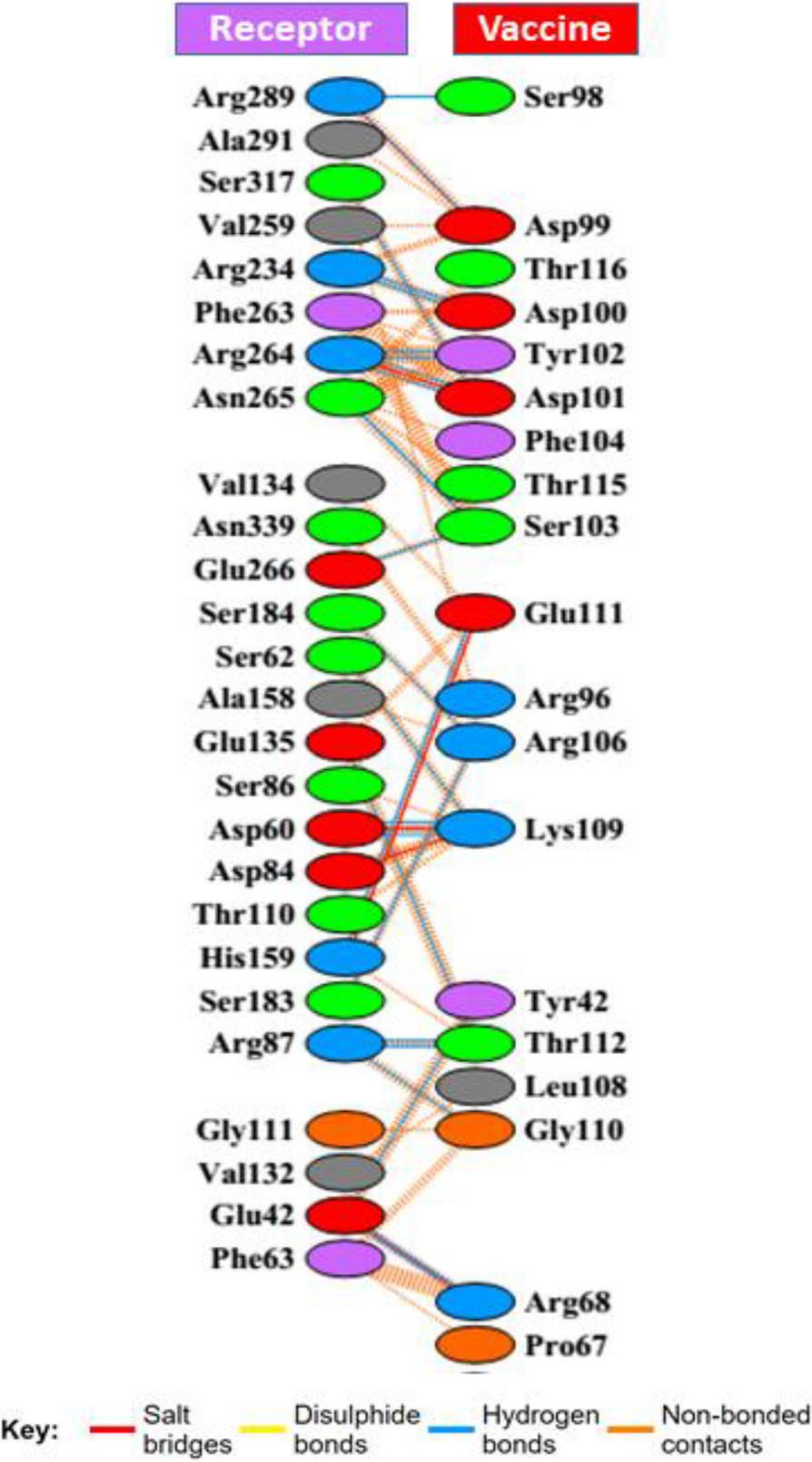
Graphical illustration of the interacting residues between the vaccine construct and the receptor. A total of 19 residues of the vaccine interacted with 26 residues of the TLR4 receptor. A number of six salt bridges (red line) and 25 hydrogen bonds (blue line) were formed in the docked complex

Docking with ClusPro 2.0 has generated a total number of 30 models with different energies. It was previously described in several studies that the model with lowest energy properly occupied the receptor. Hence, among these models, the model number 28 was found as the lowest energy model and selected for this study. The predicted vaccine was found to bind with the receptor TLR4 with an energy of − 1150.5 kcal/mol. PyMOL was used to visualize the docking interaction, shown in Fig. 6. Twenty-five hydrogen bonds and 6 salt bridges were found in the PDBsum analysis of the docked complex of vaccine and receptor (Fig. 7). Furthermore, it was discovered that the receptor contained 26 interface residues covering a surface area of 934, compared to the vaccine’s 19 interface residues, which covered a surface area of 1077. The interacting hydrogen bonds were as follows: GLU42-TYR42, GLU42-ARG68, GLU42-ARG68, ASP60-LYS109, ASP60-LYS109, SER62-LYS109, ARG87-GLY110, ARG87-THR112, ARG87-THR112, GLU135-THR112,HIS159-GLU111, SER183-ARG106, SER184-ARG106, ARG234-ASP100, ARG234-ASP100, ARG264-ASP101, ARG264-TYR102, ARG264-ASP101, ARG264-TYR102, ASN265-SER103, ASN265-SER103, GLU266-SER103, ARG289-SER98, ARG289-ASP99, and SER317-ASP101, and the chain distance was as follows: 2.95, 2.65, 2.66, 2.66, 2.66, 2.75, 2.62, 3.07,2.95, 2.96, 2.94, 2.63, 2.77, 2.74, 2.86, 2.73, 2.88, 2.67, 2.77, 2.77, 2.73, 2.79, 3.31, 2.77, and 2.90, respectively.

### MD simulation

**Fig. 8:**
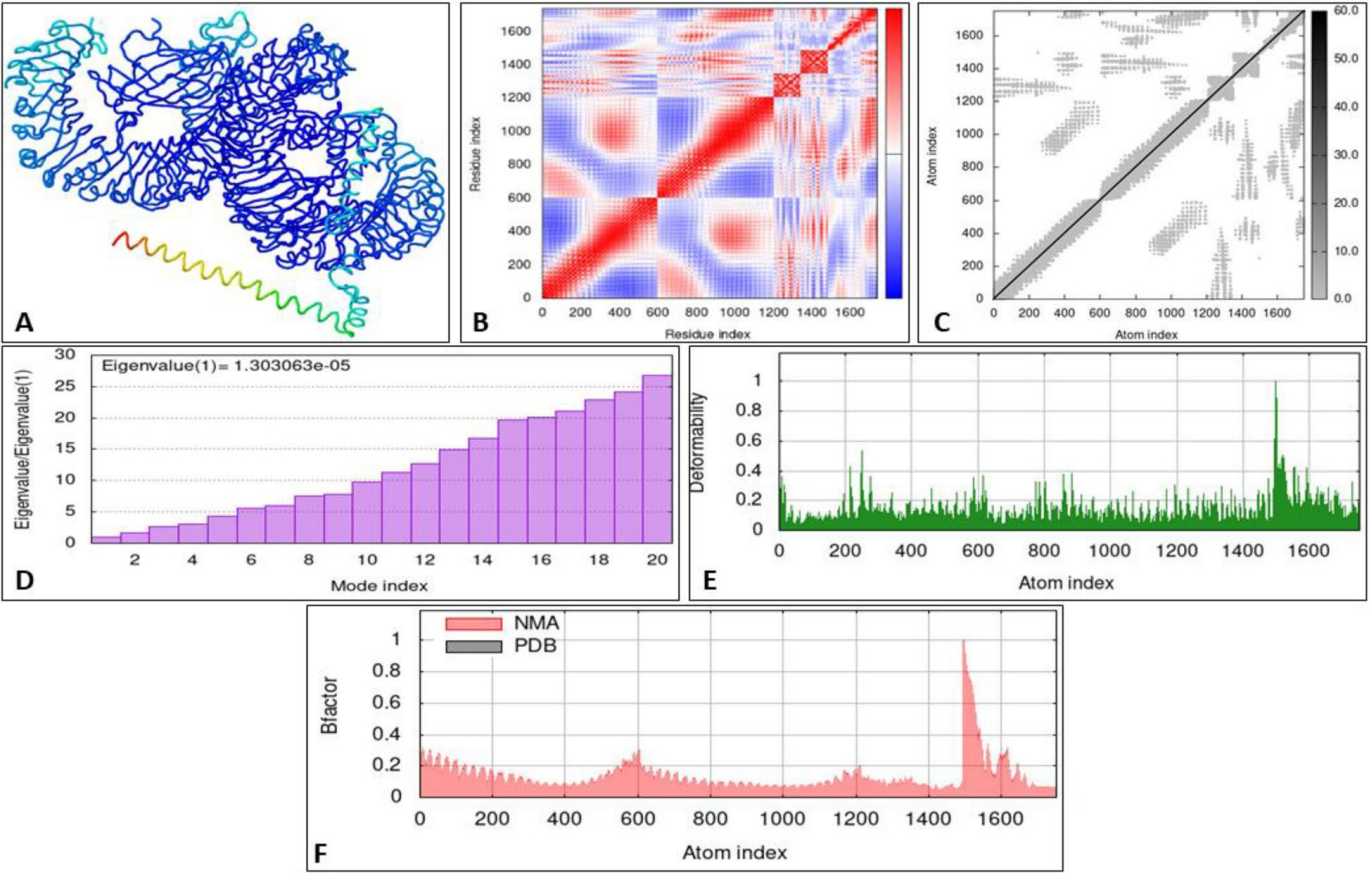
**A** Residual motion of the docking complex with TLR-4. **B** Covariance matrix analysis of the residual pair. **C** Connection spring image of the elastic network model. **D** Eigenvalue of the final complex. **E** Deformability of the final complex. **F** NMA calculation and PDB B-factor

Molecular dynamics simulation was performed to analyze the stability of the system. The final docking complex was deposited to iMODS to check the system mobility of each residue. After different analyses were performed, i.e., NMA mobility; it was confirmed that the movement of the vaccine construct is toward the TLR-4 (Fig. 8A). The eigenvalue is defined as the number proportional to the energy needed to alter the docking complex’s stability. Eigenvalue of covariance matrix analysis indicates the value of 1.303063e-05. Significantly seems to be high for deforming the complex (Fig. 8D), from the connection spring image of the elastic network model it was confirmed the presence of deformability and few hinges within the residues reflects high stability (Fig. 8E), and from NMA calculation and PDB B-factor it was confirmed lower fluctuation of the protein residue (Fig. 8F).

### C-immune simulation

**Fig. 9:**
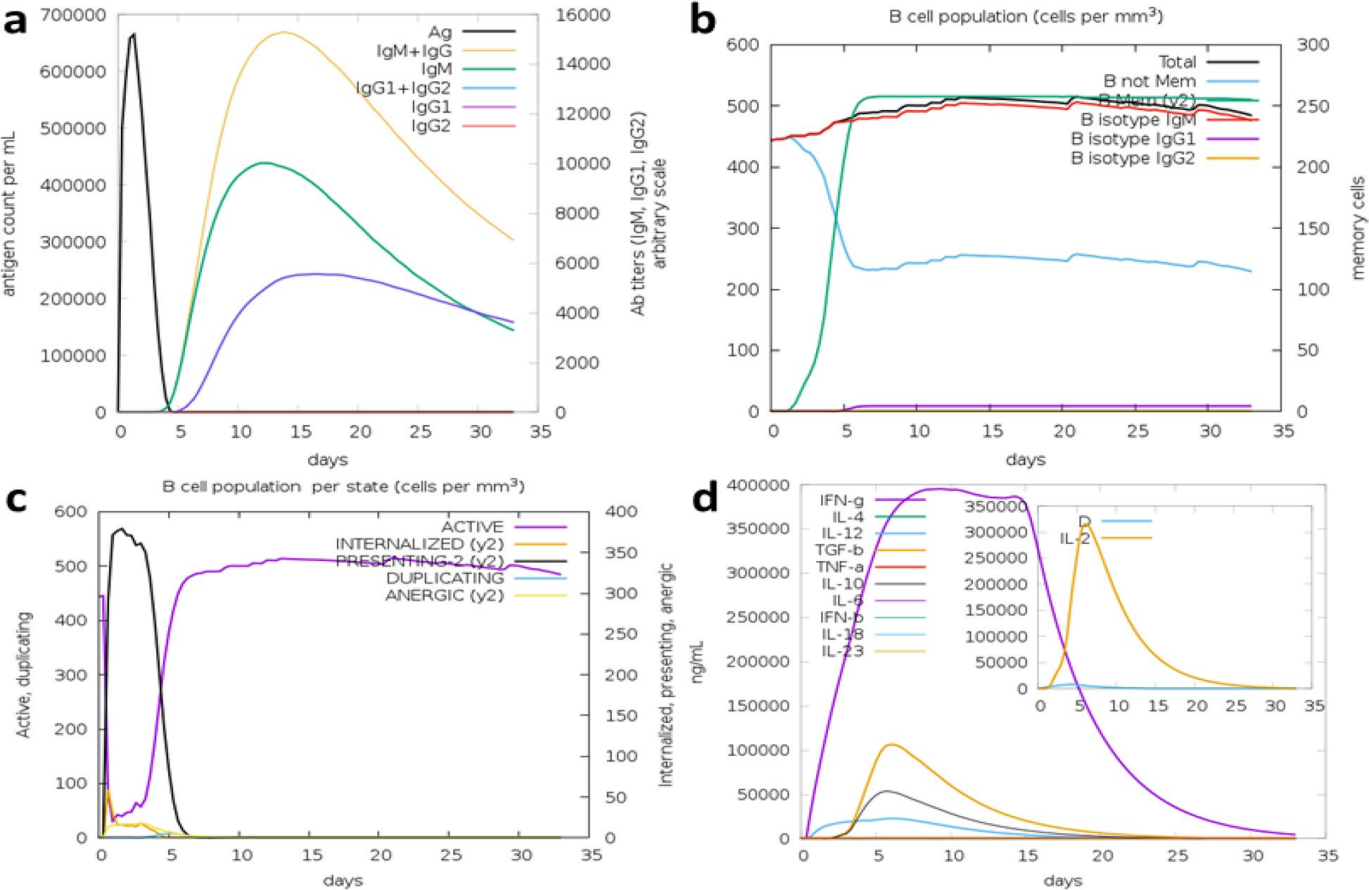
In silico immune simulation of the vaccine. **a** Production of immunoglobulin in response to antigen injection (represented in black vertical lines). **b** Prediction of B-cell population (cell per mm^3^). **c** T-helper cell population per state (cell per mm.^3^). **d** Cytokines level after the injection (main plot), IL-2 level production (insert plot)

To determine human immune responses after the injection of vaccine at various times of interval, we predicted C-immune simulation by using C-immune tool. The identification of T-cytotoxic cells, T-helper cells, antibodies production, and other aspects of the immune response that were compatible with actual immunological reactions was validated (Fig. 9). A rise in IgG1 + IgG2, IgM, and IgG + IgM was observed after the vaccine injection, which resulted in a drop in the antigen concentration (Fig. 9a & b). After the injection of vaccine, high production can be seen in Tc (cytotoxic) and natural killer cell (NK cell). Additionally, after the injection of vaccine, the production of IFN-*γ* was also increased.

### In silico cloning of the final construct

**Fig. 10:**
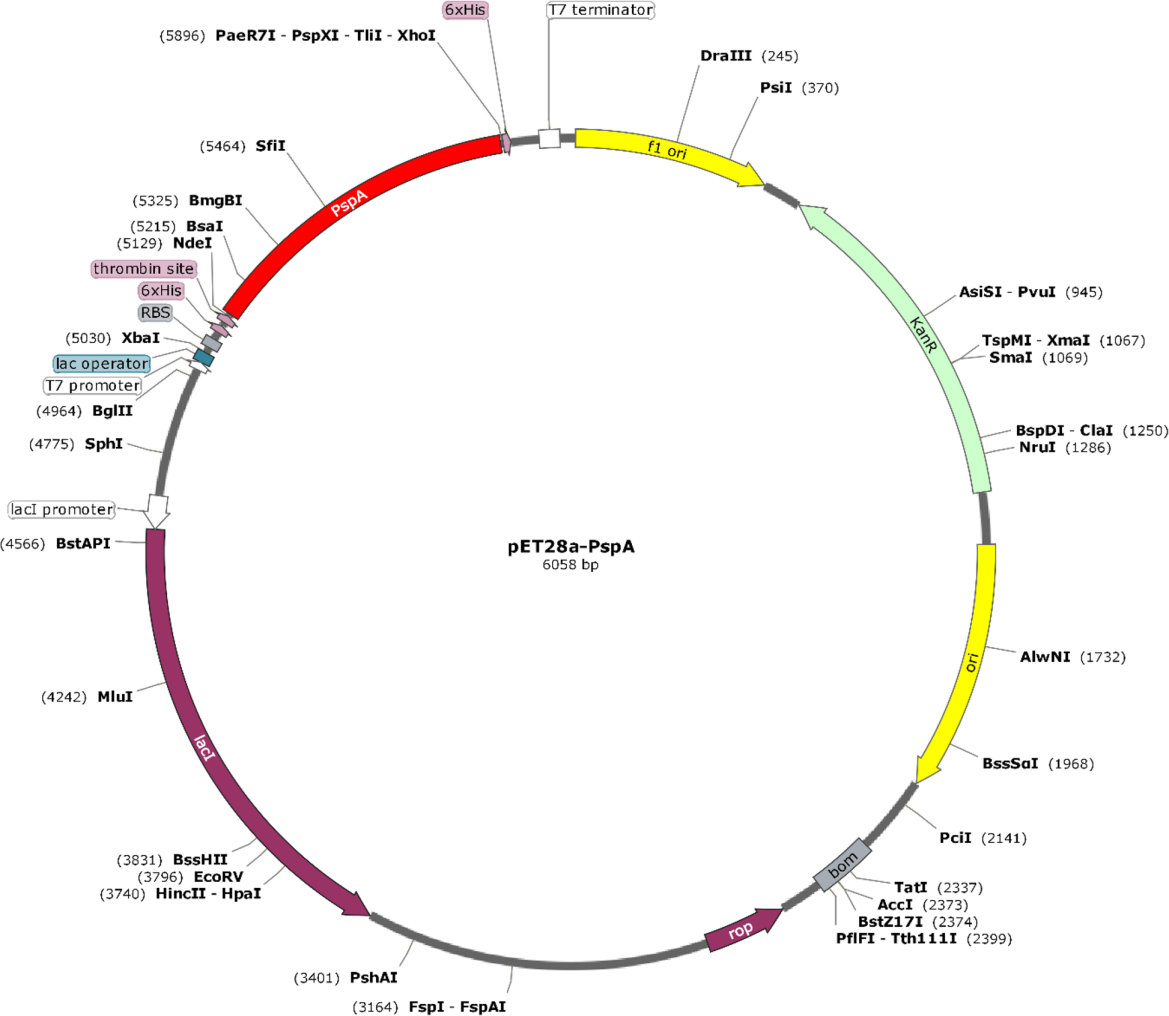
In silico cloning of the vaccine sequence into the pET28a ( +) vector, represented in red color (6058 bp). The vaccine sequence was inserted between the *Xhol* and *Ndel* restriction sites of the expression vector

In silico cloning of the engineered construct was achieved for maximum expression in the *E. coli* expression system. Codon content was diminished upon codon optimization using the JCat tool from 37 to 70%. The CAI value of the optimized sequence was 0.3296, indicating an acceptable expression probability in the *E. coli* K12 expression system. Analysis with SnapGene has identified two common sites between the expression vector pET28a ( +) and the codon-optimized vaccine sequence, including Xhol and Ndel. Both the vaccine sequence and the vector were directionally cloned into their respective cloning sites. After cloning, the final length of the vector and the insert was found to be 6058 bp. The vaccine sequence inserted in the expression vector pET28a ( +) is represented in Fig. 10.

## Discussion

The use of computational tools, particularly reverse vaccinology, is an appealing option for developing an epitomic vaccine for rapidly spreading disease. Multi-epitope-based vaccine design could be the best and most effective tool for disease control [57]. Remarkably, multi-epitope-based vaccines, which contain only peptide fragments with the highest antigen levels and the most incredible ability to elicit an immune response, represent an attractive, stable, time-saving, and inexpensive approach to vaccine development [12]. SPN is the agent responsible for causing respiratory diseases, including pneumonia, which causes severe health hazards and child deaths globally. A small number of vaccines that are currently available have severe limitations and can protect only a subset of serotypes of SPN that promote the development of pneumococcal infection. Hence, our research aimed to develop a vaccine against SPN that could produce better immunity in the host.

By utilizing several standard bioinformatics tools, the best antigenic protein and epitopes were identified to design a multi-epitope vaccine against the SPN. The vaccine was designed with three potential epitopes: CTL, HTL, and LBL. Linkers KK, CPGPG, and AAY joined these epitopes during the vaccine formulation to produce better and long-lasting protection. The critical problem of epitope vaccines is that they can quickly be broken down by proteinase in the body [12]. To circumvent this problem, the 50S ribosomal chromosome was inserted with the vaccine sequences as an adjuvant. The computational analysis of the developed vaccine revealed that it is non-allergic, has a good antigenic score (0.5722), and has good solubility expression inside *E. coli* (0.918779). To produce better immunity in the host, a vaccine needs to be stable in its performance. The instability index was computed to be 20.34, which is significantly lower than the study of Bahadori et al. (2022) [12], Dorosti et al. (2019) [14], and Dorosti, Eslami, and Negahdaripour et al. (2019) [22], showing our vaccine is highly stable in nature. The aliphatic index of 72.17 demonstrated the thermostability of the vaccine. Our constructed vaccine has a negative GRAVY index (− 0.303) and a higher solubility score (0.919) which demonstrates that our vaccine is hydrophilic and interacts with water molecules more efficiently. The GRAVY index of our designed vaccine is significantly lower than the vaccine constructed in a study by Dorosti et al. (2019) [14], and the solubility score is higher than the vaccine created by Bahadori et al. (2022) [12], Dorosti et al. (2019) [14], and Tarahomjoo and Ghaderi (2019) [21]. The Ramachandran plot represents the suitable characteristics essential for the potential vaccine structure. The Ramachandran plot data indicated that the predicted model’s stereochemical quality is suitable for further use. The next step, which is crucial in the validation of a vaccine, is molecular docking. The negative value of binding energy implies that the formation of a vaccine-receptor complex can occur spontaneously. A lower score of binding energy indicates a strong interaction between the receptor and ligand, which is essential for producing strong immunity in the host body. The docking study used in this research revealed a much lower binding energy, a large number of hydrogen bonds and interacting residues in the docked complex of constructed vaccine, and the receptor molecule TLR4. Our predicted vaccine binds with the receptor TLR4 with an energy of − 1150.5 kcal/mol, which is comparatively lower than the in silico study of Bahadori et al. (2022) [12]. Finally, MD simulation was performed by using the online tool iMODS. The immune simulation graph shows that our designed vaccine has a significant level of IgM generation after inoculation, suggesting the primary response. A rise in immunoglobulin expression in the B cell also contributed to a decrease in the antigen concentration. JCat software is used to predict the best protein expression in the *E. coli* K12 strain for codon optimization to enhance transcription and translation efficacy. The GC content of our vaccine is satisfactory as it falls in the optimal range of > 30 and < 70%, which is desirable for expression [12]. The in silico cloning in *Escherichia coli* was performed to facilitate the path of further wet laboratory experiments by other researchers to produce an effective vaccine as well as to serve as a protocol for further in silico analysis of cloning experiments. In future, our team plans to use wet lab work and the *E. coli* expression system to produce this prototype vaccine.

A large number of recent studies have used robust computational methods to select effective epitopes and design new vaccines against various pathogens, including *Staphylococcus aureus* [15], *Vibrio harveyi* [36], *Neisseria gonorrhoeae* [54], and SARS-CoV-2 [57]. Based on the methods employed in these studies, the current analysis suggests a final peptide construct as the best multi-epitope vaccination. The antigenic epitopes found in this work may also be used in subsequent research to create novel epitope-based peptide vaccines. However, though in silico studies have several limitations and a lack of reliability, the efficacy of the vaccine can be further verified by wet laboratory experiments on cell lines and animals which can be followed by clinical trials.

## Conclusion

Drug targets and scientific approaches are involved in making effective and potentially lifesaving medications. Although many drugs are available in the market, vaccines play a significant role in preventing pathogen infection. Throughout this study, we investigated a novel therapeutic vaccine against the PspA of *Streptococcus pneumoniae* by utilizing several bioinformatics tools. In this study, a multi-epitope-based vaccine was constructed using in silico tools, and its binding affinity with human cell receptor molecule (TLR4) was calculated. Analysis of molecular docking, molecular dynamic simulation, C-immune simulation, codon adaptation, and in silico cloning validated our designed compound as a suitable vaccine candidate. Our analysis has revealed that the designed vaccine in this study has the potential to generate higher immunogenicity in the host without any possible toxicity and allergenicity. Thus, our vaccine can be an attractive alternative to the conventional vaccines that are currently being used against pneumococcal disease and has several shortcomings.

## Supplementary Information

**Additional file 1:** **Supplementary Table 1.** Protein information of the 10 protein sequences obtained from NCBI by BlastP search. **Supplementary Table 2.** CTL Epitopes identified by NetCTL 1.2 server and their antigenic and allergenic properties predicted with VaxiJen and AllerTOP 2 server, respectively. **Supplementary Table 3.** HTL epitopes less than ic50 <250nm and interacting with 10 or more alleles. **Supplementary Table 4.** LBL epitopes predicted with IEDB B cell epitope prediction tool by utilizing Kolaskar & Tongaonkar Antigenicity method. 

## Data Availability

The dataset(s) supporting the conclusions of this article is (are) included within the article and in the Supplementary tables.

## Abbreviations

3D: Three-dimensional
CAI: Codon adaptation index
CbpA: Choline-binding protein A
CbpD: Choline-binding protein D
C-IMMSIM: C language version of immune system simulator
C-score: Confidence score
CTL: Cytotoxic T lymphocytes
DDCP: D-Alanyl-D-alanine-carboxypeptidase
FBP: Fibronectin-binding protein
HTL: Helper T lymphocytes
IEDB: Immune Epitope Database
IFN-γ: Interferon-*γ* (gamma)
JCat: JAVA Codon Adaptation Tool
LBL: Linear B lymphocytes
NCBI: National Center for Biotechnology Information
PDB: Protein Data Bank
PiuA: Pneumococcal iron uptake protein
PspA: Pneumococcal surface protein A
RCSB: Royal Chemical Society of Britain
SPN: *Streptococcus pneumoniae*
TLR-4: Toll-like receptor 4
