# Supplementary material for: An immunoinformatics approach to epitope-based vaccine design against PspA in Streptococcus pneumoniae
Source: J Genet Eng Biotechnol. 2023 May 11;21:57. doi: 10.1186/s43141-023-00506-9 (PMC10173237; doi:10.1186/s43141-023-00506-9)
Supplement: Supplementary file 1 — Additional file 1: Supplementary Table 1. Protein information of the 10 protein sequences obtained from NCBI by BlastP search. Supplementary Table 2. CTL Epitopes identified by NetCTL 1.2 server and their antigenic and allergenic properties predicted with VaxiJen and AllerTOP 2 server, respectively. Supplementary Table 3. HTL epitopes less than ic50 <250nm and interacting with 10 or more alleles. Supplementary Table 4. LBL epitopes predicted with IEDB B cell epitope prediction tool by utilizing Kolaskar & Tongaonkar Antigenicity method. [file 43141_2023_506_MOESM1_ESM.docx]

**Supplementary Tables:**

**Supplementary Table 1:** Protein information of the 10 protein sequences obtained from NCBI by BlastP search

| Accession No | Protein name | Sequence | VaxiJen score | Antigenicity |
| --- | --- | --- | --- | --- |
| WP_001035315.1 | pneumococcal surface protein A [Streptococcus pneumoniae] | MNKKKMILTSLASVAILGAGFVASQPTVVRAEESPVASQSKAEKDYDAAKKDAKNAKKAVEDAQKALDDAKAAQKKYDEDQKKTEEKAALEKAASEEMDKAVAAVQQAYLAYQQATDKAAKDAADKMIDEAKKREEEAKTKFNTVRAMVVPEPEQLAETKKKSEEAKQKAPELTKKLEEAKAKLEEAEKKATEAKQKVDAEEVAPQAKIAELENQVHRLEQELKEIDESESEDYAKEGFRAPLQSKLDAKKAKLSKLEELSDKIDELDAEIAKLEDQLKAAEENNNVEDYFKEGLEKTIAAKKAELEKTEADLKKAVNEPEKPAPAPETPAPEAPAEQPKPAPAPQPAPAPKPEKPAEQPKPEKTDDQQAEEDYARRSEEEYNRLTQQQPPKAEKPAPAPKTGWKQENGMWYFYNTDGSMATGWLQNNGSWYYLNSNGAMATGWLQYNGSWYYLNANGAMATGWAKVNGSWYYLNANGAMATGWLQYNGSWYYLNANGAMATGWAKVNGSWYYLNANGAMATGWLQYNGSWYYLNANGAMATGWAKVNGSWYYLNANGAMATGWVKDGDTWYYLEASGAMKASQWFKVSDKWYYVNGLGALAVNTTVDGYKVNANGEWV | 0.5847 | ANTIGEN |
| COP73186.1 | surface protein A [Streptococcus pneumoniae] | MNKKKMILTSLASVAILGAGFVASQPTFVRAEESPVASQSKAEKDYDAAVKKSEAAKKAYEEAKKALEEAKVAQKKYEDDQKKTEEKAELEKEASEAIAKATEEVQQAYLAYQRASNKAEAAKMIEEAQRRENEARAKFTTIRTTMVVPEPEQLAETKKKAEEAKAKEPKLAKKAAEAKAKLEEAEKKATEAKQKVDAEEVAPQAKIAELENQVHRLEQELKEIDESESEDYAKEGFRAPLQSKLDAKKAKLSKLEELSDKIDELDAEIAKLEDQLKAAEENNNVEDYFKEGLEKTIAAKKAELEKTEADLKKAVNEPEKPAEEPSQPEKPAEEAPAPKPEKPAEQPKAEKTDDQQAEEDYARRSEEEYNRLTQQQPPKAEKPAPAPQPEQPAPAPKTGWKQENGMWYFYNTDGSMATGWLQNNGSWYYLNSNGAMATGWLQNNGSWYYLNANGAMATGWAKVNGSWYYLNANGAMATGWLQYNGSWYYLNASGAMATGWAKVNGSWYYLNANGSMATGWLQYNGSWYYLNANGAMATGWAKVNGSWYYLNANGSMATGWVKDGDTWYYLEASGAMKASQWFKVSDKWYYVNGLGALAVNTTVDGYKVNANGEWV | 0.5701 | ANTIGEN |
| VKC73360.1 | pneumococcal surface protein PspA [Streptococcus pneumoniae] | MNKKKMILTSLASVAILGAGFVASQPTFVRAEDAPVANQSQAEKDYDAAMKKSEAAKKEYEDAKKVLAEAEADQKKYEDDQKKTEEKAEKAKAASEEIAKATEEVQKAVLDYITAIRNHNESGKKSAEEAEKKAKERETAARKKFDTIQTTIVVPEPDELAKTQKKAEEAAKNKPELTKKLEEAKVKLEEAEKKATEAKQKLDAEEVALQAKIAELEYEVQRLEKELEEINESDSEDYAKEGFRAPLQSKLDAKKAKLSKLEELSDKIVELDAEIAKLEDQLKAAEENNNVEDYFKEGLEKTVAAKKAELEKTEADLKKAVNEPEKPAPAPETPAPEAPAEQPKPAPAPQPAPAPKPEKPAEQPKPEKTDDQQAEEDYARRSEEEYNRLTQQQPPKAEKPAPAPQPEQPAPAPKTGWKQENGMWYFYNTDGSMATGWLQNNGSWYYLNSNGAMATGWLQNNGSWYYLNANGAMATGWLQNNGSWYYLNSNGAMATGWLQYNGSWYYLNASGAMATGWAKVNGSWYYLNANGSMATGWLQYNGSWYYLNANGAMATGWAKVNGSWYYLNANGSMATGWVKDGDTWYYLEASGAMKASQWFKVSDKWYYVNGLGALAVNTTVDGYKVNANGEWV | 0.5881 | ANTIGEN |
| VTG63986.1 | Pneumococcal surface protein A [Streptococcus pneumoniae] | MNKKKMILTSLASVAILGAGFVTSQPTVVRAEESPVASQSKAEKDYDAAVKKSEAAKKHYEEVKAKSEAAKKEYEDGKAAQKKYEDDQKKTEEKAEEERKASEEEQAANLKYQQELVKYIRENDPTKKAEAKKAMDEAEKEYKKKQTEFAEVRAKVIPSAEELKKTRQKAEEAKLKEAEVAKKVEETKKQEEEAKAKLEEAEKKATEAKQKVDAEEVAPQAKIAELENQVHRLEQELKEIDESDSEDYIKEGFRAPLQSELDAKQAKLSKLEELSDKIDELDAEIAKLEKDVEDFKNSNGEQAEQYRAAAEEDLAAKQAELEKTEADLKKAVNEPEKPAPAPETPAPEAPAEQPKPAPAPQPAPAPKPEKPAEQPKPEKTDDQQAEEDYARRSEEEYNRLTQQQPPKAEKPAPAPKTGWKQENGMWYFYNTDGSMATGWLQNNGSWYYLNSNGAMATGWLQNNGSWYYLNANGAMATGWLQNNGSWYYLNSNGAMATGWAKVNGSWYYLNANGSMATGWLQYNGSWYYLNANGAMATGWAKVNGSWYYLNANGSMATGWLQYNGSWYYLNANGAMATGWAKVNGSWYYLNANGSMATGWVKDGDTWYYLEASGAMKASQWFKVSDKWYYVNGLGALAVNTTVDGYEVNANGEWV | 0.6555 | ANTIGEN |
| VME33070.1 | Pneumococcal surface protein A [Streptococcus pneumoniae] | MILTSLASVAILGAGFVTSQPTVVRAEESPVASQSKAEKDYDAAVKKSEAAKKHYEEVKAKSEAAKKEYEDGKAAQKKYEDDQKKTEEKAEEERKASEEEQAANLKYQQELVKYIRENDPTKKAEAKKAMDEAEKEYKKKQTEFAEVRAKVIPSAEELKKTRQKAEEAKLKEAEVAKKVEETKKQEEEAKAKLEEAEKKATEAKQKVDAEEVAPQAKIAELENQVHRLEQELKEIDESDSEDYIKEGFRAPLQSELDAKQAKLSKLEELSDKIDELDAEIAKLEKDVEDFKNSNGEQAEQYRAAAEEDLAAKQAELEKTEADLKKAVNEPEKPAPAPETPAPEAPAEQPKPAPAPQPAPAPKPEKPAEQPKPEKTDDQQAEEDYARRSEEEYNRLTQQQPPKAEKPAPAPKTGWKQENGMWYFYNTDGSMATGWLQNNGSWYYLNSNGAMATGWLQNNGSWYYLNANGAMATGWLQNNGSWYYLNSNGAMATGWAKVNGSWYYLNANGSMATGWLQYNGSWYYLNANGAMATGWAKVNGSWYYLNANGSMATGWLQYNGSWYYLNANGAMATGWAKVNGSWYYLNANGSMATGWVKDGDTWYYLEASGAMKASQWFKVSDKWYYVNGLGALAVNTTVDGYEVNANGEWV | 0.6675 | ANTIGEN |
| VNS06961.1 | pneumococcal surface protein PspA [Streptococcus pneumoniae] | MNKKKMILTSLASVAILGAGFVASQPTFVRAEDAPVANQSQAEKDYDAAMKKSEAAKKEYEDAKKVLAEAEAAQKKYEDDQKKTEEKAEKAKAASEEIAKATEEVQKAVLDYITAIRNHNESGKKSAEEAEKKATEAKQKLDAEEVALQAKIAELEYEVQRLEKELEEINESDSEDYAKEGFRAPLQSKLDAKKAKLSKLEELSDKIVELDAEIAKLEDQLKAAEENNNVEDYFKEGLEKTVAAKKAELEKTEADLKKAVNEPEKPAPAPETPAPEAPAEQPKPAPAPQPAPAPKPEKPAKQPKPEKTDDQQAEEDYARRSEEEYNRLTQQQPPKAEKPAPAPQPEQPAPAPKTGWKQENGMWYFYNTDGSMATGWLQNNGSWYYLNSNGAMATGWLQNNGSWYYLNANGAMATGWLQNNGSWYYLNSNGAMATGWLQYNGSWYYLNASGAMATGWAKVNGSWYYLNANGSMATGWLQYNGSWYYLNANGAMATGWAKVNGSWYYLNANGSMATGWVKDGDTWYYLEASGAMKASQWFKVSDKWYYVNGLGALAVNTTVDGYEVNANGEWV | 0.5570 | ANTIGEN |
| VPY37639.1 | Pneumococcal surface protein A [Streptococcus pneumoniae] | MSESIPDVNPLLTRLKTEKDNLEAAKRQLKEAKAAEKTEKEAKVKKVETELAQAKEKVKKQAEEDRRNYPTNTYKTLELEIAESDVKVKEAELELVKEEANESRNEEKIKQAKAKVESKQAEATRLEKIKTDRKKAEEEAKVKLEEAEKKATEAKQKLDAERAKEVAPQAKIAELEYEVQRLEKALEEIDESESEDYAKEGFRAPLQSKLDAKKAKLSKLEELSDKIDELDAEIAKLEDQLKAAEENNNVEDYFKEGLEKTIAAKKAELEKTEADLKKAVNEPEKPAEEPSQPEKPAEEAPAPEQPTEPTQPEKPAEQPQPAPAPQPEKTDDQQAEEDYARKSEEAYNRLTQQQPPKTEKPAPAPKTGWKQENGMWYFYNTDGSMATGWLQNNGSWYYLNSNGAMATEWLQYNGSWYYLNASGAMATGWAKVNGSWYYLNANGAMATGWLQYNDSWYYLNASGAMATGWAKVNGSWYYLNANGAMATGWLQNNGSWYYLNANGAMATGWAKVNGSWYYLNANGSMATGWVKDGDTWYYLEASGAMKASQWFKVSDKWYYVNGLGALAVNTTVDGYTVNENGEWV | 0.6232 | ANTIGEN |
| WP_079114724.1 | unnamed protein product [Streptococcus pneumoniae] | VKLEEAEKKATEAKQKLDAERAKEVAPQAKIAELEYEVQRLEKALEEIDESESEDYAKEGFRAPLQSKLDAKKAKLSKLEELSDKIDELDAEIAKLEDQLKAAEENNNVEDYFKEGLEKTIAAKKAELEKTEADLKKAVNEPEKPAEEPSQPEKPAEEPSQPEKPAEEAPAPEQPTEPTQPEKPAEQPQPAPAPQPEKTDDQQAEEDYARKSEEAYNRLTQQQPPKTEKPAPAPQPEQPAPAPKTGWKQENGMWYFYNTDGSMATGWLQNNGSWYYLNSNGAMATEWLQYNGSWYYLNASGAMATGWAKVNGSWYYLNANGAMATGWLQYNDSWYYLNASGAMATGWAKVNGSWYYLNANGAMATGWLQNNGSWYYLNANGAMATGWAKVNGSWYYLNANGSMATGWVKDGDTWYYLEASGAMKASQWFKVSDKWYYVNGLGALAVNTTVDGYTVNENGEWV | 0.4771 | NON-ANTIGEN |
| CVP30657.1 | Pneumococcal surface protein A [Streptococcus pneumoniae] | MDAERAKEVAPQAKIAELEYEVQRLEKALEEIDESESEDYAKEGFRAPLQSKLDAKKAKLSKLEELSDKIDELDAEIAKLEDQLKAAEENNNVEDYFKEGLEKTIAAKKAELEKTEADLKKAVNEPEKPAEEPSQPEKPAEEPSQPEKPAEEAPAPEQPTEPTQPEKPAEQPQPAPAPQPEKTDDQQAEEDYARKSEEAYNRLTQQQPPKTEKPAPAPQPEQPAPAPKTGWKQENGMWYFYNTDGSMATGWLQNNGSWYYLNSNGAMATEWLQYNGSWYYLNASGAMATGWAKVNGSWYYLNANGAMATGWLQYNDSWYYLNASGAMATGWAKVNGSWYYLNANGAMATGWLQNNGSWYYLNANGAMATGWAKVNGSWYYLNANGSMATGWVKDGDTWYYLEASGAMKASQWFKVSDKWYYVNGLGALAVNTTVDGYTVNENGEWV | 0.4418 | NON-ANTIGEN |
| VFH39006.1 | Pneumococcal surface protein A [Streptococcus pneumoniae] | MDAERAKEVAPQAKIAELEYEVQRLEKALEEIDESESEDYAKEGFRAPLQSKLDAKKAKLSKLEELSDKIDELDAEIAKLEDQLKAAEENNNVEDYFKEGLEKTIAAKKAELEKTEADLKKAVNEPEKPAEEPSQPEKPAEEAPAPEQPTEPTQPEKPAEQPQPAPAPQPEKTDDQQAEEDYARKSEEAYNRLTQQQPPKTEKPAPAPQPEQPAPAPKTGWKQENGMWYFYNTDGSMATGWLQNNGSWYYLNSNGAMATEWLQYNGSWYYLNASGAMATGWAKVNGSWYYLNANGAMATGWLQYNDSWYYLNASGAMATGWAKVNGSWYYLNANGAMATGWLQNNGSWYYLNANGAMATGWAKVNGSWYYLNANGSMATGWVKDGDTWYYLEASGAMKASQWFKVSDKWYYVNGLGALAVNTTVDGYTVNENGEWV | 0.4470 | NON-ANTIGEN |

**Supplementary Table 2:** CTL Epitopes identified by NetCTL 1.2 server and their antigenic and allergenic properties predicted with VaxiJen and AllerTOP 2 server, respectively.

| **Serial No** | **Peptide** | **C-score** | **Antigenicity** | **Allergenicity** |
| --- | --- | --- | --- | --- |
| 1 | SMATGWLQY | 2.4644 | Non-antigen | Allergen |
| 2 | AMDEAEKEY | 1.7329 | 0.4003 (probable antigen) | Negative |
| 3 | AVNTTVDGY | 1.1861 | 1.1496 (probable antigen) | Allergen |
| 4 | KQENGMWYF | 0.759 | Non-antigen | Negative |
| 5 | KSEAAKKEY | 1.643 | 1.5677 (probable antigen) | Negative |
| 6 | KSEAAKKHY | 1.5182 | 1.4407 (probable antigen) | Negative |
| 7 | LQNNGSWYY | 1.9528 | Non-antigen | Allergen |
| 8 | LQYNGSWYY | 1.1261 | Non-antigen | Allergen |
| 9 | VKDGDTWYY | 1.8125 | 0.7789 (probable antigen) | Allergen |
| 10 | WAKVNGSWY | 0.9714 | Non-antigen | Allergen |
| 11 | WLQNNGSWY | 1.5838 | Non-antigen | Allergen |
| 12 | WLQYNGSWY | 1.4974 | Non-antigen | Allergen |
| 13 | WVKDGDTWY | 1.0013 | Non-antigen | Negative |
| 14 | YARRSEEEY | 0.8104 | 1.0156 (probable antigen) | Allergen |

**Supplementary Table 3:** HTL epitopes less than ic50 <250nm and interacting with 10 or more alleles

| **S.N.** | **Epitopes** | **Interacting alleles** | **Antigenicity** | **Allergenicity** |
| --- | --- | --- | --- | --- |
| 1 | AGFVTSQPTVVRAEE | HLA-DRB1*09:01, HLA-DRB1*04:01, HLA-DRB3*02:02, HLA-DRB1*07:01, HLA-DRB1*07:01, HLA-DRB1*01:01, HLA-DRB3*01:01, HLA-DRB1*13:02, HLA-DRB5*01:01, HLA-DRB1*04:05, HLA-DRB1*15:01 | 0.4282 (Probable ANTIGEN ) | Negative |
| 2 | DTWYYLEASGAMKAS | HLA-DQA1*01:02/DQB1*06:02, HLA-DRB1*07:01, HLA-DQA1*05:01/DQB1*03:01, HLA-DRB1*13:02, HLA-DRB1*01:01, HLA-DRB1*04:05, HLA-DRB5*01:01, HLA-DRB1*11:01, HLA-DRB1*09:01, HLA-DRB1*04:01, HLA-DRB3*01:01 | 0.4952 ( Probable ANTIGEN ) | Positive |
| 3 | GAGFVTSQPTVVRAE | HLA-DRB1*09:01, HLA-DRB1*04:01, HLA-DRB3*02:02, HLA-DRB1*07:01, HLA-DRB1*01:01, HLA-DRB3*01:01, HLA-DRB1*13:02, HLA-DQA1*05:01/DQB1*03:01, HLA-DRB5*01:01, HLA-DRB1*04:05, HLA-DRB1*15:01 | 0.7378 ( Probable ANTIGEN ) | Positive |
| 4 | GDTWYYLEASGAMKA | HLA-DRB1*07:01, HLA-DQA1*01:02/DQB1*06:02, HLA-DRB1*13:02, HLA-DQA1*05:01/DQB1*03:01, HLA-DRB1*01:01, HLA-DRB1*04:05, HLA-DRB5*01:01, HLA-DRB1*11:01, HLA-DRB3*01:01, HLA-DRB1*09:01, HLA-DRB1*04:01 | 0.4586 ( Probable ANTIGEN ) | Positive |
| 5 | GSWYYLNANGAMATG | HLA-DRB1*13:02, HLA-DQA1*01:02/DQB1*06:02, HLA-DQA1*05:01/DQB1*03:01, HLA-DRB1*04:05, HLA-DRB5*01:01, HLA-DRB1*15:01, HLA-DRB1*01:01, HLA-DRB1*11:01, HLA-DRB3*02:02, HLA-DRB1*04:01, HLA-DRB1*09:01, HLA-DRB3*01:01, HLA-DRB1*07:01 | 0.6041 ( Probable ANTIGEN ) | Negative |
| 6 | GSWYYLNANGSMATG | HLA-DRB1*13:02, HLA-DQA1*05:01/DQB1*03:01, HLA-DRB1*01:01, HLA-DRB1*04:01, HLA-DRB1*04:05, HLA-DRB1*07:01, HLA-DRB1*09:01, HLA-DRB1*11:01, HLA-DRB1*13:02, HLA-DRB3*01:01, HLA-DRB3*02:02, HLA-DRB5*01:01 | 0.7186 ( Probable ANTIGEN ) | Positive |
| 7 | GSWYYLNSNGAMATG | HLA-DRB1*13:02, HLA-DQA1*01:02/DQB1*06:02, HLA-DQA1*05:01/DQB1*03:01, HLA-DRB1*01:01, HLA-DRB1*04:01, HLA-DRB1*04:05, HLA-DRB1*07:01, HLA-DRB1*09:01, HLA-DRB1*11:01, HLA-DRB1*13:02, HLA-DRB1*15:01, HLA-DRB3*01:01, HLA-DRB3*02:02, HLA-DRB5*01:01 | 0.5133 ( Probable ANTIGEN ) | Negative |
| 8 | ILGAGFVTSQPTVVR | HLA-DRB1*09:01, HLA-DRB1*07:01, HLA-DQA1*05:01/DQB1*03:01, HLA-DRB1*04:01, HLA-DRB3*02:02, HLA-DRB3*01:01, HLA-DRB1*01:01, HLA-DRB1*13:02, HLA-DRB1*04:05, HLA-DRB5*01:01 | 0.5829 ( Probable ANTIGEN ) | Negative |
| 9 | KVNGSWYYLNANGSM | HLA-DRB1*04:01, HLA-DRB1*01:01, HLA-DRB1*04:05, HLA-DRB1*11:01, HLA-DRB5*01:01, HLA-DRB3*02:02, HLA-DRB3*01:01, HLA-DRB1*09:01, HLA-DRB1*13:02, HLA-DRB1*07:01 | 0.7413 ( Probable ANTIGEN ) | Positive |
| 10 | KWYYVNGLGALAVNT | HLA-DQA1*05:01/DQB1*03:01, HLA-DRB1*12:01, HLA-DRB1*04:05, HLA-DQA1*01:02/DQB1*06:02, HLA-DPA1*03:01/DPB1*04:02, HLA-DRB1*07:01, HLA-DRB1*01:01, HLA-DRB1*09:01, HLA-DRB1*04:01, HLA-DRB5*01:01, HLA-DRB1*11:01, HLA-DRB3*02:02, HLA-DRB1*13:02, HLA-DPA1*01:03/DPB1*02:01 | 0.4330 ( Probable ANTIGEN ) | Negative |
| 11 | LGAGFVTSQPTVVRA | HLA-DRB1*09:01, HLA-DRB1*04:01, HLA-DRB1*07:01, HLA-DRB3*02:02, HLA-DRB1*01:01, HLA-DRB3*01:01, HLA-DQA1*05:01/DQB1*03:01, HLA-DRB1*13:02, HLA-DRB1*04:05, HLA-DRB5*01:01, HLA-DRB1*15:01 | 0.6418 ( Probable ANTIGEN ) | Positive |
| 12 | NGSWYYLNANGSMAT | HLA-DQA1*05:01/DQB1*03:01, HLA-DRB1*01:01, HLA-DRB1*04:01, HLA-DRB1*04:05, HLA-DRB1*07:01, HLA-DRB1*09:01, HLA-DRB1*11:01, HLA-DRB1*13:02, HLA-DRB3*01:01, HLA-DRB3*02:02, HLA-DRB5*01:01 | 0.5537 ( Probable ANTIGEN ) | Positive |
| 13 | NGSWYYLNSNGAMAT | HLA-DRB1*01:01, HLA-DRB1*04:01, HLA-DRB1*04:05, HLA-DRB1*07:01, HLA-DRB1*09:01, HLA-DRB1*11:01, HLA-DRB1*13:02, HLA-DRB1*15:01, HLA-DRB3*01:01, HLA-DRB3*02:02, HLA-DRB5*01:01 | 0.3931 ( Probable NON-ANTIGEN ) | Negative |
| 14 | NNGSWYYLNSNGAMA | HLA-DRB1*01:01, HLA-DRB1*04:01, HLA-DRB1*04:05, HLA-DRB1*07:01, HLA-DRB1*09:01, HLA-DRB1*11:01, HLA-DRB1*13:02, HLA-DRB1*15:01, HLA-DRB3*01:01, HLA-DRB3*02:02, HLA-DRB5*01:01 | 0.1754 ( Probable NON-ANTIGEN ) | Negative |
| 15 | SWYYLNANGAMATGW | HLA-DRB3*02:02, HLA-DQA1*01:02/DQB1*06:02, HLA-DRB1*13:02, HLA-DQA1*05:01/DQB1*03:01, HLA-DRB5*01:01, HLA-DRB1*01:01, HLA-DRB1*11:01, HLA-DRB1*04:05, HLA-DRB1*04:01, HLA-DRB1*09:01, HLA-DRB3*01:01, HLA-DRB1*07:01 | 0.6068 ( Probable ANTIGEN ) | Negative |
| 16 | SWYYLNANGSMATGW | HLA-DRB1*13:02, HLA-DRB1*13:02, HLA-DQA1*01:02/DQB1*06:02, HLA-DQA1*05:01/DQB1*03:01, HLA-DRB1*01:01, HLA-DRB1*04:01, HLA-DRB1*04:05, HLA-DRB1*07:01, HLA-DRB1*09:01, HLA-DRB1*11:01, HLA-DRB3*01:01, HLA-DRB3*02:02, HLA-DRB5*01:01 | 0.6594 ( Probable ANTIGEN ) | Positive |
| 17 | SWYYLNSNGAMATGW | HLA-DRB1*13:02, HLA-DQA1*01:02/DQB1*06:02, HLA-DQA1*05:01/DQB1*03:01, HLA-DRB1*01:01, HLA-DRB1*04:01, HLA-DRB1*04:05, HLA-DRB1*07:01, HLA-DRB1*09:01, HLA-DRB1*11:01, HLA-DRB3*01:01, HLA-DRB3*02:02, HLA-DRB5*01:01 | 0.5160 ( Probable ANTIGEN ) | Negative |
| 18 | TWYYLEASGAMKASQ | HLA-DQA1*05:01/DQB1*03:01, HLA-DQA1*01:02/DQB1*06:02, HLA-DRB1*07:01, HLA-DRB1*13:02, HLA-DRB1*01:01, HLA-DRB5*01:01, HLA-DRB1*04:05, HLA-DRB1*11:01, HLA-DRB1*09:01, HLA-DRB1*04:01, HLA-DRB3*01:01 | 0.7340 ( Probable ANTIGEN ) | Negative |
| 19 | VNGSWYYLNANGSMA | HLA-DRB1*01:01, HLA-DRB1*04:01, HLA-DRB1*04:05, HLA-DRB1*07:01, HLA-DRB1*09:01, HLA-DRB1*11:01, HLA-DRB1*13:02, HLA-DRB3*01:01, HLA-DRB3*02:02, HLA-DRB5*01:01 | 0.7109 ( Probable ANTIGEN ) | Positive |
| 20 | WYYLEASGAMKASQW | HLA-DQA1*05:01/DQB1*03:01, HLA-DQA1*01:02/DQB1*06:02, HLA-DRB1*07:01, HLA-DRB1*13:02, HLA-DRB5*01:01, HLA-DRB1*01:01, HLA-DRB1*11:01, HLA-DRB1*09:01, HLA-DRB1*04:01, HLA-DRB3*01:01 | 0.6924 ( Probable ANTIGEN ) | Positive |
| 21 | WYYLNANGAMATGWA | HLA-DRB3*02:02, HLA-DQA1*01:02/DQB1*06:02, HLA-DRB1*13:02, HLA-DQA1*05:01/DQB1*03:01, HLA-DRB1*01:01, HLA-DRB1*09:01, HLA-DRB1*11:01, HLA-DRB5*01:01, HLA-DRB1*04:01, HLA-DRB3*01:01 | 0.7728 ( Probable ANTIGEN ) | Negative |
| 22 | WYYLNANGSMATGWL | HLA-DQA1*01:02/DQB1*06:02, HLA-DQA1*05:01/DQB1*03:01, HLA-DRB1*01:01, HLA-DRB1*04:01, HLA-DRB1*09:01, HLA-DRB1*11:01, HLA-DRB1*13:02, HLA-DRB3*01:01, HLA-DRB3*02:02, HLA-DRB5*01:01 | 0.6401 ( Probable ANTIGEN ) | Negative |
| 23 | WYYLNSNGAMATGWA | HLA-DRB3*02:02, HLA-DQA1*01:02/DQB1*06:02, HLA-DRB1*13:02, HLA-DQA1*05:01/DQB1*03:01, HLA-DRB5*01:01, HLA-DRB1*01:01, HLA-DRB1*09:01, HLA-DRB1*11:01, HLA-DRB1*07:01, HLA-DRB1*04:01, HLA-DRB3*01:01 | 0.6820 ( Probable ANTIGEN ) | Negative |
| 24 | WYYLNSNGAMATGWL | HLA-DRB3*02:02, HLA-DQA1*01:02/DQB1*06:02, HLA-DRB1*13:02, HLA-DQA1*05:01/DQB1*03:01, HLA-DRB5*01:01, HLA-DRB1*01:01, HLA-DRB1*09:01, HLA-DRB1*11:01, HLA-DRB1*07:01, HLA-DRB1*04:01, HLA-DRB3*01:01 | 0.4967 ( Probable ANTIGEN ) | Negative |
| 25 | WYYVNGLGALAVNTT | HLA-DQA1*05:01/DQB1*03:01, HLA-DRB1*12:01, HLA-DRB1*04:05, HLA-DQA1*01:02/DQB1*06:02, HLA-DRB1*07:01, HLA-DRB1*01:01, HLA-DRB1*09:01, HLA-DPA1*03:01/DPB1*04:02, HLA-DRB5*01:01, HLA-DRB3*02:02, HLA-DRB1*13:02 | 0.3814 ( Probable NON-ANTIGEN ) | Positive |
| 26 | YYLEASGAMKASQWF | HLA-DQA1*05:01/DQB1*03:01, HLA-DQA1*01:02/DQB1*06:02, HLA-DRB1*09:01, HLA-DRB1*07:01, HLA-DRB1*04:01, HLA-DRB1*13:02, HLA-DRB1*11:01, HLA-DRB5*01:01, HLA-DRB1*01:01, HLA-DRB3*01:01 | 0.4174 ( Probable ANTIGEN ) | Positive |

**Supplementary Table 4:** LBL epitopes predicted with IEDB B cell epitope prediction tool by utilizing Kolaskar & Tongaonkar Antigenicity method.

| **S.N.** | **No.** | **Start** | **End** | **Peptide** | **Length** | **Antigenicity** | **Allergenicity** | **Toxicity** |
| --- | --- | --- | --- | --- | --- | --- | --- | --- |
| 1 | 4 | 103 | 115 | ANLKYQQELVKYI | 13 | Non-antigen | Negative | Non-toxin |
| 2 | 5 | 144 | 156 | FAEVRAKVIPSAE | 13 | Non-antigen | Negative | Non-toxin |
| 3 | 10 | 278 | 286 | AEIAKLEKD | 9 | Non-antigen | Allergen | Non-toxin |
| 4 | 21 | 614 | 636 | QWFKVSDKWYYVNGLGALAVNTT | 23 | Non-antigen | Negative | Non-toxin |
| 5 | 2 | 42 | 48 | DAAVKKS | 7 | 1.7312 | Negative | Non-toxin |
| 6 | 3 | 55 | 61 | YEEVKAK | 7 | 1.5194 | Allergen | Non-toxin |
| 7 | 14 | 461 | 466 | WYYLNA | 6 | 1.1522 | Negative | Non-toxin |
| 8 | 20 | 601 | 607 | WYYLEAS | 7 | 1.0143 | Negative | Non-toxin |
| 9 | 6 | 170 | 178 | LKEAEVAKK | 9 | 0.987 | Allergen | Non-toxin |
| 10 | 7 | 204 | 219 | KQKVDAEEVAPQAKIA | 16 | 0.9701 | Negative | Non-toxin |
| 11 | 11 | 333 | 339 | PAPAPET | 7 | 0.8689 | Negative | Non-toxin |
| 12 | 12 | 342 | 364 | PEAPAEQPKPAPAPQPAPAPKPE | 23 | 0.7306 | Negative | Non-toxin |
| 13 | 9 | 249 | 269 | RAPLQSELDAKQAKLSKLEEL | 21 | 0.6634 | Allergen | Non-toxin |
| 14 | 16 | 520 | 526 | SWYYLNA | 7 | 0.6079 | Negative | Non-toxin |
| 15 | 18 | 560 | 566 | SWYYLNA | 7 | 0.6079 | Negative | Non-toxin |
| 16 | 15 | 497 | 505 | VNGSWYYLN | 9 | 0.5862 | Allergen | Non-toxin |
| 17 | 17 | 537 | 545 | VNGSWYYLN | 9 | 0.5862 | Allergen | Non-toxin |
| 18 | 19 | 577 | 585 | VNGSWYYLN | 9 | 0.5862 | Allergen | Non-toxin |
| 19 | 1 | 4 | 35 | TSLASVAILGAGFVTSQPTVVRAEESPVASQS | 32 | 0.5177 | Negative | Non-toxin |
| 20 | 13 | 403 | 410 | AEKPAPAP | 8 | 0.479 | Negative | Non-toxin |
| 21 | 8 | 221 | 231 | LENQVHRLEQE | 11 | 0.4128 | Allergen | Non-toxin |
